# Protein bio-corona: critical issue in immune nanotoxicology

**DOI:** 10.1007/s00204-016-1797-5

**Published:** 2016-07-20

**Authors:** Monica Neagu, Zoi Piperigkou, Konstantina Karamanou, Ayse Basak Engin, Anca Oana Docea, Carolina Constantin, Carolina Negrei, Dragana Nikitovic, Aristidis Tsatsakis

**Affiliations:** 1 Immunology Department, “Victor Babes” National Institute of Pathology, Bucharest, Romania; 2 Faculty of Biology, University of Bucharest, Bucharest, Romania; 3 Laboratory of Biochemistry, Biochemistry, Biochemical Analysis and Matrix Pathobiology Research Group, Department of Chemistry, University of Patras, Patras, Greece; 4 Foundation for Research and Technology-Hellas (FORTH)/Institute of Chemical Engineering Sciences (ICE-HT), Patras, Greece; 5 Laboratório de Bioquímica e Biologia Cellular de Glicoconjugados, Programa de Glicobiologia, Instituto de Bioquímica Médica Leopoldo De Meis and Hospital Universitário Clementino Fraga Filho, Universidade Federal do Rio de Janeiro, Rio de Janeiro, Brazil; 6 Department of Toxicology, Faculty of Pharmacy, Gazi University, Ankara, Turkey; 7 Department of Toxicology, Faculty of Pharmacy University of Medicine and Pharmacy Craiova, Craiova, Romania; 8 Department of Toxicology, Faculty of Pharmacy, “Carol Davila” University of Medicine and Pharmacy, Bucharest, Romania; 9 Laboratory of Anatomy-Histology-Embryology, Medical School, University of Crete, Heraklion, Greece; 10 Department of Toxicology and Forensic Sciences, Medical School, University of Crete, Heraklion, Greece

**Keywords:** Bio-corona, Nanoparticle, Immunological identity

## Abstract

With the expansion of the nanomedicine field, the knowledge focusing on the behavior of nanoparticles in the biological milieu has rapidly escalated. Upon introduction to a complex biological system, nanomaterials dynamically interact with all the encountered biomolecules and form the protein “bio-corona.” The decoration with these surface biomolecules endows nanoparticles with new properties. The present review will address updates of the protein bio-corona characteristics as influenced by nanoparticle’s physicochemical properties and by the particularities of the encountered biological milieu. Undeniably, bio-corona generation influences the efficacy of the nanodrug and guides the actions of innate and adaptive immunity. Exploiting the dynamic process of protein bio-corona development in combination with the new engineered horizons of drugs linked to nanoparticles could lead to innovative functional nanotherapies. Therefore, bio-medical nanotechnologies should focus on the interactions of nanoparticles with the immune system for both safety and efficacy reasons.

## Introduction

The evolvement of the immune system during human’s phylogeny and ontogeny was accomplished through exposure to different chemical, physical and biological agents. All the immune mechanisms have been adapting to human environments through the course of history (Danilova 2013). Thus, there is a constant pressure from pathogens on the human immune system, an illuminating example being host–pathogen coevolution of myeloid Fc-alpha receptor 1 (FcaR1), mediating phagocytosis of opsonized pathogens and the selection of bacterial decoy staphylococcal superantigen-like 7 (SSL7) of Staphylococcus aureus that evolved to inhibit phagocytosis (Danilova 2008).

When aggressors reach the organism, the first defense layer is the physical barrier that is constituted by skin and mucosae. These layers can make use of constitutive defense weapons like cutaneous/mucosal secretion, reflexes like nociceptive reflexes or defensive vomiting to rapidly get rid of any harmful aggressors. When this barrier is breached, pathogens or injuries that threaten the body homeostasis are subjected to the action of the immune system. Two different branches of the immune system work together to restore homeostasis: the innate and adaptive immune systems. These sentinels monitor vertebrate tissues and use different tactics to recognize and overcome threats. These two branches are inherently different as regarding the sensors and mechanisms employed in order to provide either immediate protection with broad specificity (innate immunity) or delayed and prolonged protection with exquisite specificity (adaptive immunity). Furthermore, the two branches must effectively coordinate a response and physiologically down-regulate its intensity in order to prevent excessive or inappropriately targeted inflammation (Janeway 1992).

During our evolution, as humans we were exposed to food and environmental particles, a myriad of them being in the nanoparticle ranges. Airborne nanoparticles have cohabitated with humans throughout the evolutionary process, but the exposure has significantly increased in the last 100 years owing to industrial revolution (Oberdörster et al. 2005).

Nanomaterials are small particles with corresponding large surface area which confers to these corpuscles properties desirable for utilization in nanomedicine. The fact that nanomaterials have the same size range as biomolecules and cellular structures has an important duality. Thus, nanoparticles (NPs) can reach intra-cellular structures that were previously accessible only to biological aggressors. On the other hand, NPs reaching subcellular structures can be therapeutics in the rapidly evolving nanomedicine field, where applications rely on nanoscale interactions. This concept, however, is not so simple as in biological milieu, NPs interact with molecules that will completely alter their initial chemical structure (Fadeel et al. 2015).

In a physiological environment, the NP–protein corona complex formation involves absorption of protein molecules at the interfacial region between NPs and their surroundings. Understanding the corona formation process is crucial in predicting NP behavior in biological systems, including applications for nanotoxicology and development of drug delivery platforms at the nanolevel (Shaw et al. 2012; Sisco et al. 2014). Importantly, there is a dynamic interaction between NPs and biomolecular species and other chemical and organic matter which ultimately results in biological corona formation.

The resulting conformational structure of the bio-corona, which is critically dependent on intrinsic NP properties, may induce alterations in extracellular matrix (ECM) nature. Noteworthy, ECM constituents interact with cell surface receptors, growth factors and cytokines, leading to numerous signaling cascades which are closely related to cell behavior (Afratis et al. 2012; Bouris et al. 2015; Gialeli et al. 2011). Glycosaminoglycans (GAGs) are widely known for their roles in ECM remodeling and disease progression. As the most investigated ECM polysaccharides, they are indicated as effective pharmaceutical targets. Heparin, heparan sulfate and their mimetics, due to their anti-metastatic profile, are the protagonists in the GAG-based anticancer therapy (Karamanos and Tzanakakis 2012; Mizumoto and Sugahara 2013; Theocharis et al. 2015). Heparin has the highest negative charge among GAGs. This feature renders heparin responsible for its interactions with a variety of growth factors and cytokines, such as fibroblast growth factor (FGF), epidermal growth factor (EGF), insulin growth factor (IGF) and tissue necrosis factor alpha (TNF-α), allowing them to influence cancer cells’ functions, such as invasion and migration and crucial for the initiation and progression of metastasis, epithelial-to-mesenchymal transition (EMT) (Afratis et al. 2012). Taking advantage of the expanded use of NPs and heparins in effective drug delivery, it has been recently reported that two nanoheparin analogues, the nano-Styela, isolated from sea squirt *Styela plicata*, and the nanomammalian, isolated from the porcine intestine, proved to be important inhibitory agents of breast cancer, as they inhibited cell proliferation, invasion, proteasome activity and also achieved to regulate apoptosis and the expression of major matrix macromolecules (Piperigkou et al. 2016a). Based on this knowledge, it is deemed of great importance to develop applications in a frame of bio-corona–NPs complex and GAGs, especially the promising heparins and their mimics.

Although the bio-corona is a highly dynamic structure and its composition changes over time, human serum albumin (HSA), immunoglobulin (IgG) and fibrinogen were found to be the major hard corona proteins binding firmly on to the NP surfaces and showing distinctive stability (Mahmoudi et al. 2011a, b; Monopoli et al. 2012). A recent study revealed that rapid human plasma corona formation on silica and polystyrene NPs of various size and surface functionalization significantly affected NP uptake (Tenzer et al. 2013). Protein interactions with a nanoscaled surface can disrupt its native conformation, compromising thus the protein function (Li et al. 2013). Therefore, interpreting the interaction of nanomaterials with biological systems by taking into account the physicochemical properties of NPs will lead to a better understanding of bio-corona formation. Indeed, the major parameters of NPs that influence bio-corona composition include: material, size, shape, curvature, surface charge, solubility, surface functionalization and the route of administration to the body. Importantly, these properties also affect NPs distribution and their therapeutic effects (Lundqvist et al. 2011; Monopoli et al. 2012; Salvati et al. 2013).

First of all, NP size is critical factor in determining the affinity of protein binding. To note, the surface curvature has an important role in protein absorption and corona composition (Lundqvist et al. 2008). Computational and experimental studies revealed that absorbed proteins at the curved surface of NPs undergo less conformational changes than proteins adsorbed at flat surfaces of the same material (Mahmoudi et al. 2011a; Rahman et al. 2013). Moreover, it has been demonstrated that NPs of the same surface charge but of different size absorb proteins with different affinities (Deng et al. 2009). For instance, gold NPs of larger size were coated with a thicker layer of absorbed proteins as compared to gold NPs of smaller size (Dobrovolskaia et al. 2009). NPs surface charge and non-electrostatic residue-specific interactions are another important factor that affects protein corona composition. A recent study revealed that the increased surface charge of NP resulted in higher protein absorption to bio-corona. Specifically, positively charged NPs absorb proteins with isoelectric point lower that 5.5, whereas negatively charged NPs absorb proteins with an isoelectric point higher than 5.5 (Aggarwal et al. 2009). Moreover, bio-corona proteins are threatened by denaturation according to NPs surface charge. For instance, positively or negatively charged gold NPs denature proteins. On the other hand, neutral NP surface constituents do not affect proteins structure (Lundqvist et al. 2008).

Another important parameter for NPs–bio-corona interactions is the hydrophilicity/hydrophobicity that is correlated with the surface charge. In general, hydrophilic NPs present decreased plasma protein absorption than hydrophobic NPs with the same affinity (Cedervall et al. 2007a). This results in more available protein-binding sites on the surfaces of hydrophobic copolymers (Lindman et al. 2007; Saha et al. 2014). Moreover, a recent study demonstrated that plasma proteins exhibit enhanced affinity to hydrophobic NP domains. For example, cholesterol-free liposomes bind more proteins than cholesterol-rich liposomes (Dos Santos et al. 2007; Karmali and Simberg 2011).

Up to date, there are limited bibliographic data as regards the role of surface functionalization in protein absorption. It was shown that non-functionalized NPs cannot agglomerate grapheme, whereas, conversely, PEGylated NPs have increased affinity to immune-competent proteins as compared to albumins (Aggarwal et al. 2009; Pozzi et al. 2014). Recent study that assesses several parameters in the protein absorption by gold NPs revealed that the total amount of protein binding was governed only by the molecular weight of PEG coating (Dobrovolskaia et al. 2014). In summary, understanding the influence of NPs to bio-corona formations will be a key for safe and efficient design and application of NPs.

Exploiting the dynamic process of protein bio-corona development in combination with the new engineered horizons of drugs linked to nanoparticles could provide innovative functional nanomedicine approaches. Engineered NPs paired with a bio-corona have already been utilized both in vitro and in rodent in vivo models for pharmacokinetic purposes (Eliasof et al. 2013). Before proceeding to human clinical trials, many physiological factors must be seriously considered, such as the nature of corona proteins and, as regards the species used in vivo, their basal metabolic rate, their circulation blood levels and importantly their size. Indeed, the importance of the body surface area has already been established, showing the allometric relation of the conjugation and the cellular uptake (Eliasof et al. 2013). Based on this theory, concerns are raised regarding the distribution and the evolution of NPs–bio-corona complexes inside the human body, because of human body size and the circulation time needed (Sahneh et al. 2015).

## Nanoparticles characteristics influencing bio-corona composition

Nanoparticles interactions with biomolecules are influenced by a number of various factors, with additional effects regarding the structure of the ensuing bio-corona. In that context, given the place for protein adsorption (i.e., the NP–direct environment interface), it stands to reason that protein corona formation should depend on physical and chemical characteristics of both NPs and their biological surroundings (Piperigkou et al. 2016b). Protein adsorption on NP surface is therefore influenced by such determinants as NP components and configuration (Roach et al. 2005; Maiorano et al. 2010), their hydrophobic/hydrophilic characteristics (Walczyk et al. 2010) and sedimentation (particularly in systems of in vitro exposure) (Dutta et al. 2007), whereas as regards the surrounding NP environment, one may mention among others factors such as the temperature, pH and incidence of certain functional groups.

### NP influencing factors

#### NP components and configuration

Nanoparticle components and configuration and the resulting surface chemistry have a key influence as to which particular proteins may bind to NPs as well as on their specific affinities. According to a study conducted in that respect, focused on the specificity of the binding of proteins in human plasma to metal oxide NPs available on the market (e.g., zinc oxide, silicon dioxide and titanium dioxide) under conditions of similar surface charge, titanium and silicon dioxide NPs adsorb related proteins (e.g., alpha-2-acid glycoprotein, apolipoprotein D, clusterin) which differ considerably from those adsorbed by zinc oxide NPs (haptoglobin-alpha, Ig heavy chain alpha, transferrin) (Deng et al. 2009).

#### NP size

Studies have been conducted which show the key role of NP size to determine the type of proteins adsorbed to NP surfaces, thereby influencing bio-corona composition (Lundqvist et al. 2008; Vertegel et al. 2004). Indicative is the study by Lundqvist et al. (2008) conducted on polystyrene NPs of sizes varying between 50 and 100 nm, demonstrating discrete protein corona formation when these NPs were exposed to human plasma. Furthermore, an investigation of the influence of silica NP size on lysozyme adsorption revealed the determining role of NP size in establishing adsorbed lysozyme structure and function (Vertegel et al. 2004). Moreover, NP size is an important determinant of NP surface curvature, the latter strongly influencing the composition and conformation of adsorbed to corona proteins (Lynch and Dawson 2008). The characteristic, as compared to bulk materials, NP surface high curvature determines protein-binding affinities distinct from those of bulk materials similar in composition (Hill et al. 2009). In actual terms, NP highly curved surfaces are characterized by lower inter-protein interactions, which results in marked differences in protein corona composition. Inter-protein interactions represent the interplay mediated by electrostatic forces and/or molecular docking between proteins, phenomenon that can take place in the protein bio-corona (De Las and Fontanillo 2010). Characteristically, in the same material, the number and frequency of conformational changes are lower in proteins adsorbed at NP highly curved surface in comparison with those occurring in proteins adsorbed at flat surface areas (Lynch et al. 2011; Rahman et al. 2013). For instance, in studies of protein corona formation of silica NPs of differing size upon exposure to blood plasma (Tenzer et al. 2013), it has been shown that NP size significantly influenced protein binding and corona composition of proteins identified, even in cases of particle size variations as low as 10 nm. In the same manner, larger silica NPs were found to adsorb such proteins as prothrombin or gelsolin (regulating actin), whereas lipoprotein clusterin was shown to bind to small silica NPs. Further studies along the same lines had, however, disclosed no correlation between adsorption of proteins such as actin or immunoglobulin (IgG) and NP size (Tenzer et al. 2013).

An additional study investigating interactions between human plasma and incubated colloidal gold 30- and 50-nm-sized NPs had shown adsorption of a greater range of proteins on the smaller gold NPs as compared to the larger ones. This may be attributed to the multi layered interactions, as when a cationic protein binds anionic gold surface at one site, it results in the positioning of another anionic protein at an alternative site (Dobrovolskaia et al. 2009). More recently, the effect of NP size on the binding and Hill constants was investigated (Lacerda et al. 2009), focusing on gold NPs (5–100 nm) incubated in ordinary human blood proteins (albumin, γ-globulin, fibrinogen, insulin and histone). Consequently, the study found a significant correlation between the size of studied NPs and both the binding (K) and the Hill constants, more specifically the former constant increasing and the latter decreasing with NP size. At the same time, the thickness of the protein corona is also directly dependent on NP size, as is conformational change.

Hemocompatibility is another important issue in the protein bio-corona field. Coronas developed on silica and polystyrene NPs were constituted, in less than a half minute, of more than 300 different proteins. The complexity and rapidity of corona formation can induce hemolysis, thrombocyte activation, Nuptake and endothelial cell death (Tenzer et al. 2013). On single-walled carbon nanotubes, human serum proteins comprising the corona bind competitively and exhibit altered cellular interaction pathways which results in a reduced cytotoxicity of coated NPs (Ge et al. 2011). More recent studies have shown that the composition of the protein corona did not correlate with NP hematocompatibility; hence, when assessing hematotoxicity, new updated tests should be put in use (Dobrovolskaia et al. 2014).

#### NP shape

Nanoparticle shape as well plays an important role, strongly influencing the general biological responses to the NP and more specifically the manner of protein adsorption onto NP surface. Studies carried out in that respect have revealed an impressive effect of gold NP shape on their interactions with the various layers of the cell and, in particular, the more pronounced associations of cells with spherical-shaped than with rod-shaped gold NPs (Cox et al. 1999). The same selective effect of NP shape on protein binding has been shown in a study on titanium dioxide NPs, i.e., spherical NPs were unique in displaying apolipoprotein D and clusterin, whereas no such proteins were identified on rod- or tube-shaped NPs (Deng et al. 2009).

#### NP modifying by functional groups/coating

For undesirable protein absorption, mechanisms can be put in place to either prevent or control composition of the protein corona. One such mechanism is to provide the NP surface with certain functions, through incorporation of various chemical groups, which “hide” it from the “sight” of immune cells. A second similar mechanism would be the coating of the NP surface with polymers as polyethylene glycol-PEG, in practice known as PEGylation, meant to prevent NP recognition by the reticuloendothelial system (RES) and reduce protein binding. By inhibiting the generation of the biomolecular corona, a remarkably rapid transport of NPs across the endothelium can be achieved. One added advantage of this approach is the possibility to control PEG density on NP surface, allowing for longer circulation in the blood stream. Alternatively to PEG, silicon may also be used as a coating agent, exhibiting the same beneficial effect on protein adsorption (Gref et al. 2000; Perry et al. 2012; Engin et al. 2015). In practice, studies have been conducted in order to examine the abilities of various coating agents to provide control over NP–protein interaction. For instance, the action of polystyrene NPs provided with various functional groups on cultures of endothelium cells was studied (Ehrenberg et al. 2009). These authors concluded that the ability of NP surfaces to adsorb protein is a marker of their propensity to interact with cells as well as that the identity of bound proteins does not influence cell–NP association. The utilization of various coating agents (PEG, Pluronic F127, poloxamer, dextran, poly(oxyethylene), polysorbate and poloxamine) enhanced control over various aspects of NP–protein interactions including protein binding and NP bio-distribution (Aggarwal et al. 2009).

For instance, one study targeted the action of polystyrene NPs provided with a surface functionalized with various functional groups on endothelium cell cultures showing the possibility of control on NP–cell interaction as well as the lack of influence of bound protein identity on the cell–NP association (Ehrenberg et al. 2009). Studies in that respect also included polystyrene nanospheres coated with poloxamine 908. This approach revealed a reduced adsorption of fibronectin to coated NPs in this manner in comparison with coat-free similar nanospheres (Moghimi et al. 1993). Likewise, pluronic F127 coating of both amorphous silica particles and single-walled carbon nanotubes was demonstrated to increase NP dispersion and significantly diminish serum protein adsorption (Dutta et al. 2007).

#### NP surface charge

The NP surface charge is an important determining factor in the composition of the protein corona as well as in the evolution of the respective biological system. Thus, opsonins readily recognize positively charged NPs, leading to their removal by the RES and formation of deposits at the liver and spleen level (Mahmoudi et al. 2011a, b). To prevent such detrimental occurrences, the coating of the NP surface with negatively charged groups (with the effect of a 30–50 mV range negative zeta physiological potential) is a feasible option. On exposure of coated NPs to the biological environment, proteins adsorbed on the respective surface induce marked decrease in zeta potential to negative, 5–10 mV (Ehrenberg et al. 2009), which shows the direct relation between the protein corona nature and the colloidal stability of such complexes. In a separate study, conducted on gold NPs provided with negative, neutral and positive ligands, it has been demonstrated that charged ligands (irrespective of charge) induce protein distortion, whereas neutral ligands allow preservation of protein structure (Lynch and Dawson 2008). Moreover, upon investigating the surface charge density impact of negatively charged polymeric NPs, it was found that an increase in NP surface charge density leads to increased absorption of plasma protein (Gessner et al. 2002). Furthermore, it has been demonstrated in the case of polystyrene NPs that proteins <5.5 in isoelectric points are preferably adsorbed on the positively charged surfaces, in contrast to proteins >5.5 in isoelectric points which have an intrinsic affinity to negatively charged NPs charged.

#### NP hydrophilicity/hydrophobicity

In addition to protein adsorption capacities being higher to NPs with a hydrophobic surface as compared to NPs bearing hydrophilic surfaces, the former NPs have a higher capacity to distort proteins adsorbed at their surface and induce loss of their original structure (Roach et al. 2005). Similarly, in a separate study, it has been shown that in the formation of the protein corona, hydrophobic NPs have an affinity for apolipoprotein binding, in contrast to hydrophilic NPs, which display a typical adsorption preference for fibrinogen, IgG and albumin (Cedervall et al. 2007a; Gessner et al. 2000). Moreover, the direct relation is reinforced by study findings showing increase in protein stoichiometry with elevation of hydrophobicity (Cedervall et al. 2007b), as well as shorter albumin residence time on hydrophobic than hydrophilic particles with a concomitant higher coverage of hydrophobic particle surface at the point of equilibrium.

### Biological environment factors

The components and organization of the biological setting where the NP–protein interaction is perpetrated constitute an additional group of key factors which influence the composition of the protein corona (Nel et al. 2009; Roach et al. 2005). A study was therefore undertaken and conducted in various biological environments in order to demonstrate this impact. The study was performed by incubating gold NPs with citrate caps of varying sizes, in commonly used cellular media including Roswell Park Memorial Institute medium (RPMI) and Dulbecco modified Eagle’s medium (DMEM) to which fetal bovine serum—FBS—was added, all only differing in amino acid, glucose and salt content (Maiorano et al. 2010). In continuation, the DMEM- and RPMI-mediated corona formation on the gold NPs was analyzed by a number of techniques, leading to a range of revealing conclusions such as: a significant dependence on time in the case of DMEM-mediated formation of protein corona, on one hand, and a reduction, accompanied by specific dynamics, of RPMI protein corona.

Human epithelial cervical cancer as well as a human leukemic monocyte lymphoma cell line (U937) was treated with 15-nm gold NPs in both DMEM and RPMI media, and in continuation, various viability assays were performed. This approach revealed substantial differences in cellular uptake, dynamics and NP–protein complexes bio-distribution. Thus, in RPMI-cultured cells, NP–protein complexes were internalized to a markedly higher extent as compared to DMEM-cultured cells, leading to enhanced cytotoxic effects. Furthermore, RPMI-induced protein corona was inferior in abundance and stability compared to the protein corona formed in the DMEM culture (Maiorano et al. 2010). Thus, these authors conclude that in dynamic extracellular environments, the original biological identity may become altered, and with it cellular uptake. Last but not least, mass spectroscopy and sodium dodecyl sulfate-polyacrylamide gel electrophoresis (SDS-PAGE) used to characterize NP–protein complexes showed that composition of the protein corona is not related to serum protein amounts (Maiorano et al. 2010). All the above differences in NP–protein complexes show that cellular response is affected by both NP and NP environmental-specific features, which points to the importance of in-depth assessment of the two elements to more accurately determine their putative interactions.

#### Other factors

In addition to specificities of the biological milieu and the NP in question, one must not rule out the potential action of several other less obvious factors such as plasma concentration, gradient plasma, temperature and composition of the cell membrane, acting at the level of the bio-nanoparticle interface, whose capacity to determine the protein corona composition and the ensuing cellular response must not be overlooked. All the above emphasize the need for comprehensive research to elucidate all influencing factors and thus allow for better formulation of nanomedicines, prevention of undesirable events and thus development of efficient high-quality nanotherapeutics.

## New immunological identity of nanoparticles

The bio-corona entity as vehicle for immunological identity has been recently introduced. NPs interacting with biological systems have a surface corona of biomolecules that may dictate their biological behavior. Hence, 2 years ago the concept of “synthetic identity” characterizing the material’s intrinsic properties which can trigger the “biological identity” sustained mainly by the bio-corona components was launched. The complex characteristics of the bio-corona will determine the interactions of NPs with cells and tissues. Therefore, the duality stated in introduction, nanotoxicology versus nanomedicine, relies actually to a large extent on the bio-corona characteristics (Fadeel et al. 2013). NPs can interact with proteins, membranes, cells, DNA and organelles and “generate” a nanoparticle–biological interface. These are complex interactions that depend on colloidal forces as well as dynamic bio-physicochemical characteristics of the particle. The protein bio-corona will cover the nanoparticle, will define the intra-cellular uptake pathway and will in the end generate local, but as well as systemic processes balancing the thin thread of biocompatibility versus bioadverse effects (Nel et al. 2009).

**Fig. 1:**
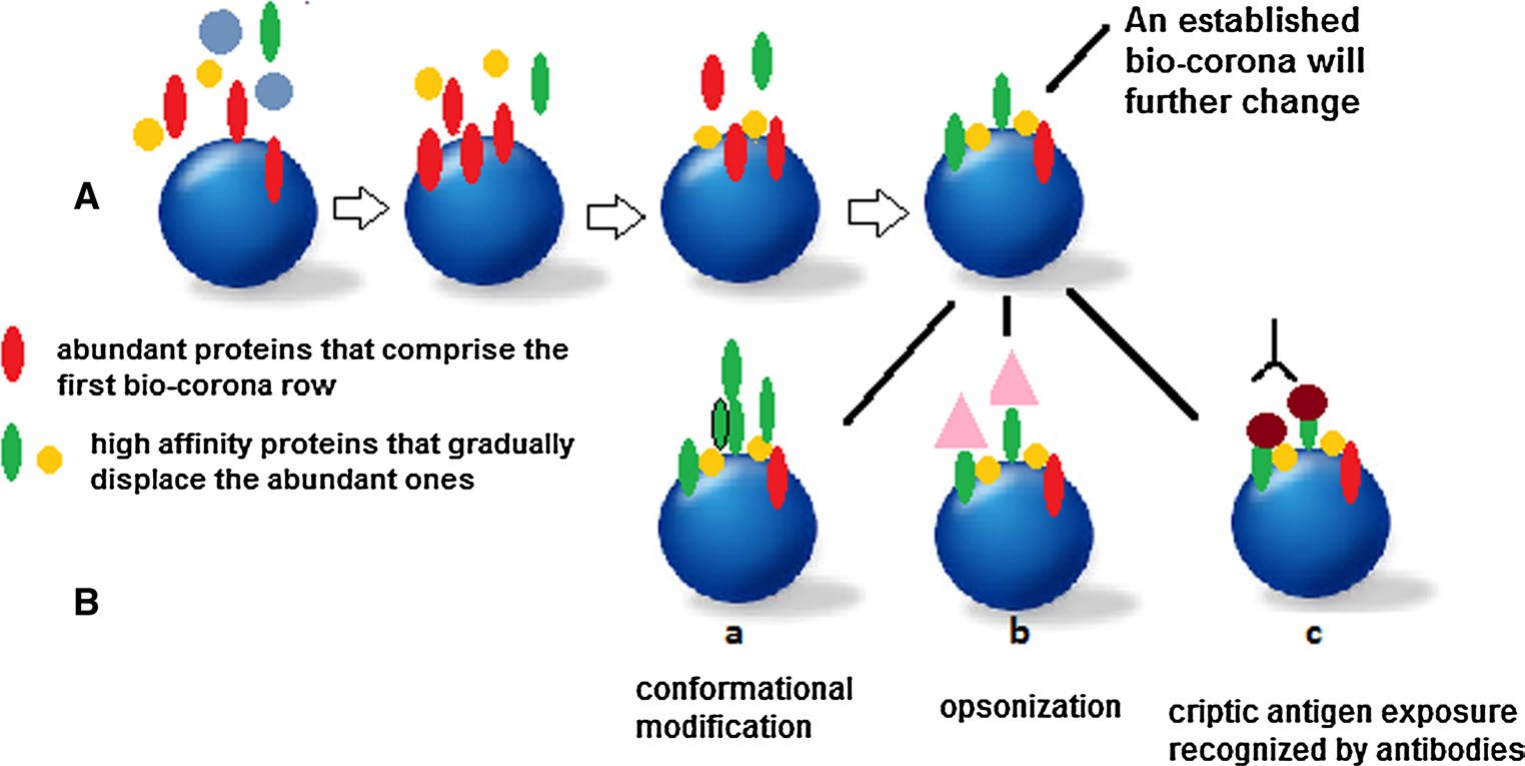
Bio-corona dynamics—**A** Chemical–physical characteristics of the particle induce the formation of the corona in a biological environment. Proteins of different affinities reach the nanoparticle; the abundant particles (*red dots*) take the *first row* but are gradually displaced by higher-affinity proteins (*green* and *yellow dots*); **B** an established bio-corona will change its composition due to protein crowding and conformational changes (*a*), opsonization (*b*) and exposure of cryptic antigens that trigger antibodies interaction (*c*). In the end, a new bio-corona profile will appear, and hence, other biological effects are initiated (colour figure online)

The initial characteristics of NP define the first level of interactions with biological molecules (Fig. 1). The characteristic of protein attachment/detachment rates, various competitive biological binding interactions, steric hindrance induced by different polymeric structures and the protein profile of the body fluid(s) makes a *dynamic* bio-corona. The corona can change when particles move from one biological compartment to another, e.g., passing through cellular membrane to other intra-cellular compartments. Potential changes in protein structure and function as a result of interacting with the NP surface can lead to potential molecular mechanisms of injury that could contribute to disease pathogenesis.

Several groups have been lately studying the mathematical models to predict bio-corona formation (Li et al. 2013). A mathematical model that describes the dynamics of nanoparticle corona complex formation was actually published. The model shows two phases of corona complex dynamics. In the first phase, there is a rapid protein binding to the free surface of NPs. During the second phase, there is a continuous association and dissociation of protein molecules. Thus, in the biological environment, the NPs slowly modulate the composition of their bio-corona. Given sufficient time, composition of the corona complex reaches an equilibrium state of stable composition. This recently suggested model shows that the dynamics of bio-corona formation constitute a vital aspect of interactions between NPs and living organisms. Moreover, authors pinpoint that modeling could better predict behavior for in vivo systems (Sahneh et al. 2013).

Continuing in this direction, the same group has recently shown that toxicology testing in animal models is extremely important. Indeed, assessing the impact of bio-corona kinetics on expected tissue distribution of NPs across species has shown that the potential fate of NPs in vivo is dependent upon basal metabolic rate. In other words, while engineered NPs can successfully reach target cells in rodent models, the results may be different in humans because an increased circulation time allows for further bio-corona evolution (Sahneh et al. 2015). From this point of view, *time* and *space do matter*. In other words, the chances for the immune system to detect a coated NP increase progressively with time.

**Fig. 2:**
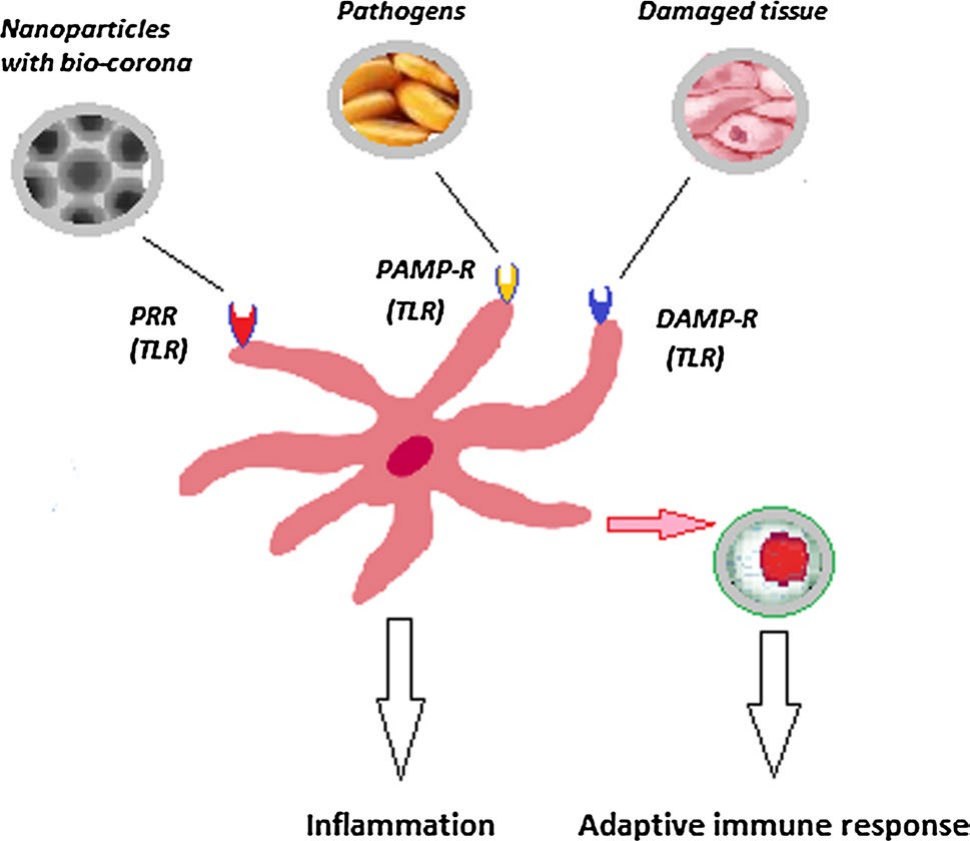
Dendritic cell (DC)—key figure in immune activation. Receptors on DC recognize damage-associated molecular patterns (DAMP), pathogen-associated molecular patterns (PAMPs) and nanoparticle-associated molecular patterns (NAMPs) with pattern recognition receptors (PRR) like the Toll-like receptors family. Upon recognition, DCs can trigger direct inflammatory response, but by activating, T cells can likewise trigger adaptive immunity (adapted after Fadeel 2012)

As indicated in the introduction, after breaching the chemical–physical barrier, the innate immune system is the first line of defense. Cells of the innate immune branch are ready for microbial invasion as they are capable to recognize pathogen-associated molecular patterns (PAMPs) using pattern recognition receptors. The immune system also responds to the altered self, namely to tissue damage, a process triggered by the so-called danger- or damage-associated molecular patterns (DAMPs) or “alarmins” (Fadeel 2012) (Fig. 2).

In addition to the self–non-self immune recognition, in the early 1994, the “danger” hypothesis was developed. In this aspect, the immune system is more concerned with entities that do damage than with those that are foreign. Thus, the primary *driving force* is the need to detect and protect against danger focusing more on *endogenous* than on exogenous signals. The cues represent the alarm signal that originates from an injured tissue (Matzinger 1994).

**Fig. 3:**
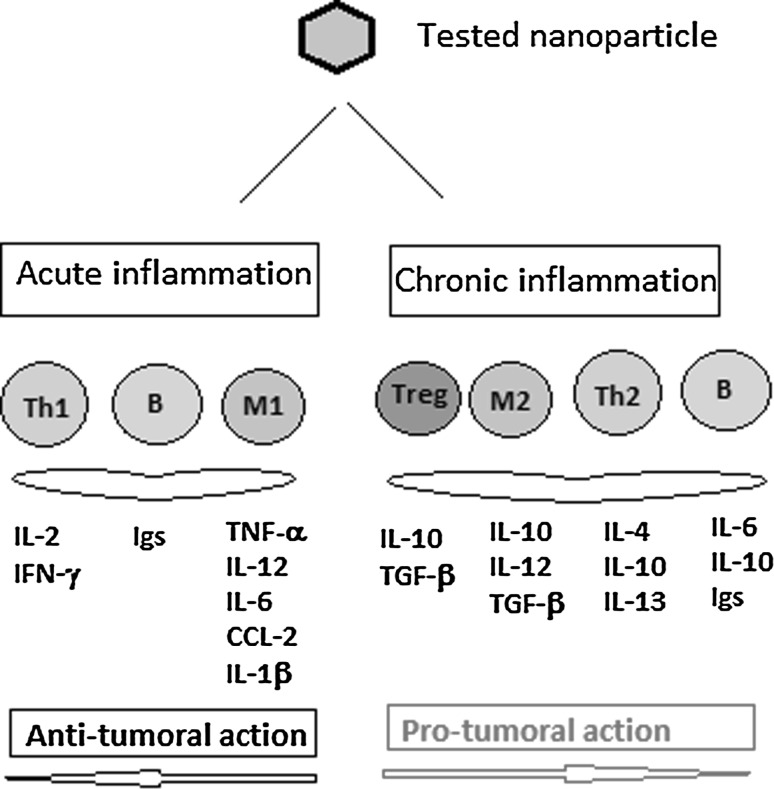
Depending on the bio-corona composition, the same nanoparticle can develop immune patterns that sustain pro- or anti-tumoral activities

In the danger model, many of the PAMPs and DAMPs alarm signals may belong to an evolutionarily ancient alert system in which the *hydrophobic portions* of biological molecules act, when exposed, as universal signals of damage to initiate immunity (Fadeel 2012). NPs can act as danger signals because pathogens display PAMPs and damaged tissues release DAMPs that act as a secreted alarmin; thus, engineered NPs coated with bio-corona of complex protein structure can act as nanomaterial-associated molecular patterns (NAMPs). These molecular signatures are recognized by pattern recognition receptors (PRRs), including innate immunity Toll-like receptors. The activation of PRRs triggers inflammation and alerts the adaptive immune system to an imminent danger. Thus, NPs coated with bio-corona, displaying hydrophobic surfaces, are interpreted as danger signals by the immune system. Indeed, Moyano et al. (2012) have shown in animal models that nanoparticle hydrophobicity dictates immune responses. These authors demonstrate that the gene expression profiling of mouse splenocytes exposed ex vivo to gold NPs is altered. Actually, the immune cells are probably “blind” or at least “short sighted” to the naked NP surfaces (Moyano et al. 2012), while the bio-corona composition can initiate alternative immune patterns (Fig. 3). Thus, if the bio-corona composition can activate the components of the immune system like helper T lymphocytes type 1 (Th1), B lymphocytes and macrophages type 1 (M1), the entire panel of secreted molecules, starting with Ig, cytokines and chemokines, will generate an acute inflammation reaction but not a prolonged one and hence no neoplastic events. On the other hand, if the bio-corona activates Th2, M2 and regulatory T lymphocytes (Tregs), then the array of secreted molecules will sustain a chronic inflammation and hence possible pro-tumoral activity (Farrera and Fadeel 2015). Therefore, balancing of these two pathways is of utmost importance when NPs are intended for nanomedicine use.

Importantly, *Nature* has its own ways to try to bypass the action of immune cells. Thus, spores of the human opportunistic fungal pathogen *Aspergillus* *fumigatus* are surrounded by a natural protein corona of hydrophobin, making them “invisible” to cells of the immune system (Aimanianda et al. 2009). Using this property, hydrophobin-functionalized porous silicon NPs were shown to display a pronounced change in the degree of plasma protein adsorption in vitro and altered biodistribution in vivo when compared to uncoated NPs. This study provides further evidence that “stealth” properties can be engineered by manipulating the bio-corona on NPs (Sarparanta et al. 2012).

### Bio-corona inducing innate immunity

Unless they are specifically designed to avoid it, NPs are rapidly covered, in contact with biological fluids, by a selected group of biomolecules to form a corona that interacts with biological systems. As shown above, NP act as scaffold for biomolecules, which adsorb rapidly to the NPs’ surface and confer a new biological identity to the respective NPs (Monopoli et al. 2012). The dynamics of bio-corona formation constitute vital aspect of interactions between NPs and living organisms. Initially, proteins rapidly bind to the free surface of NPs. During the second phase, continuous association and dissociation of protein molecules with NPs slowly change the composition of the corona complex. Finally, composition of the corona complex reaches an equilibrium state of stable composition (Sahneh et al. 2013). Hence, proteins compete for the NP surface, generating a protein corona that is largely defined by the biological identity of the particle. In this respect, the knowledge of rates, affinities and stoichiometries of protein association with or dissociation from NPs is important for understanding the nature of the immunological response to the particles by the functional machinery of cells (Cedervall et al. 2007a). In some cases, the protein corona may not fully saturate the surface area of nanoparticle and instead bind at discreet sites, leaving much of NP surface unobstructed, which could also affect its toxicity (Rybak-Smith and Sim 2011). Bio-corona may extend the half-life of the NPs in systemic circulation by preventing the non-specific uptake by cells of RES. Noteworthy, over seventy different serum proteins may heterogeneously be adsorbed to the surface of NPs. Variations in serum protein adsorption correlate with differences in the mechanism and efficiency of NP uptake by a macrophage cell (Walkey et al. 2012). Nuclear factor-kappaB (NF-кB)-dependent cytokine production in THP-1 monocyte cell line was one of the first reports regarding immune nanotoxicology, but the actual set of cytokines are different from one study to the other due to the different protein orientation and/or folding leading to different receptor activation and hence cytokine release (Deng et al. 2011), and, as presented in Fig. 3, different bio-corona compositions can portray discrete immune patterns.

The mononuclear phagocytic systems’ capabilities can be affected by the adsorption of surfactants or coatings protein, as in the case of uncoated nanospheres which form a complex with fibronectin and can be taken up by hepatic Kupffer cells (Dutta et al. 2007). In this manner, the number of binding sites on a NP is determined by the size of the particle as well as by the identity of the corona proteins. However, the hydrophobicity of the NP strongly influences its protein-binding properties (Cedervall et al. 2007a; Lindman et al. 2007). Biopersistent engineered nanomaterials activate innate immune responses via inflammasomes in macrophages, triggering IL-1beta release and neutrophilic infiltration (Thompson et al. 2014). On the other hand, proteins coated on the surface of NPs may undergo conformational changes, resulting in the exposure of new epitopes which could be presented as antigen by antigen-presenting cells to initiate adaptive immune response (Nel et al. 2009). The NP surface may induce abnormal unfolding of the bound proteins to form novel conformational epitopes or may also induce unfolding of the native protein structure to expose hidden epitopes (Saptarshi et al. 2013). Such occult epitopes may affect the functionality of the bound proteins and lead to an unwanted immune response. Deng et al. (2011) showed that negatively charged poly(acrylic acid)-conjugated gold NPs bound fibrinogen from blood plasma and induced its unfolding, which in turn activated the receptor macrophage-1 antigen (Mac-1) on THP-1 cells, causing release of inflammatory cytokines via the NF-κB pathway (Deng et al. 2011).

When performing a detailed identification of plasma protein binding to copolymer NPs, Cederval’s group in 2009 showed that HSA, despite its higher concentration that is reflected by initially more abundant binding, is rapidly replaced by the higher-affinity and slower-exchanging apolipoproteins AI, AII, AIV and E. Furthermore, high-density lipoproteins (HDLs) bind to copolymer NPs with much higher affinity than other lipoproteins, probably mediated by apolipoprotein A-I, which is its major component. In brief, together with the alternate protein-binding patterns in the corona, copolymer NPs bind complete HDL complexes and may be recognized by living systems as HDL complexes (Hellstrand et al. 2009). Albumin is the major protein adsorbed onto single-walled carbon nanotubes, whereas the targeting of albumin to scavenger receptors is well known. Targeting of the scavenger receptor pathway by a single-walled carbon nanotubes—albumin—complex could result in interference with the innate immune response (Dutta et al. 2007). However, while common proteins can bind to different NPs, the biological outcome may not be the same (Deng et al. 2013). Both protein’s structure and NP type contribute to bio-corona fate during intra-cellular uptake by target cells. Firstly, serum proteins are adsorbed onto the surface of NPs and protein structure can be modified during this step. For instance, a change in the secondary structure of BSA adsorbed on cationic polystyrene NPs forwards the protein–NP complex to scavenger receptors of the target cell. By contrast, when NP is an anionic polystyrene type, the adsorbed BSA preserves its natural structure, causing the binding of BSA–NPs complex to albumin receptors of target cells (Fleischer and Payne 2014). On negatively charged gold NPs, HSA has different behavior in relation to NPs concentration. At lower NPs concentrations, there is a higher affinity for tryptophan, whereas at higher concentrations, both tryptophan and tyrosine residues participated in the interaction, without binding to the free sulfhydryl groups (Mariam et al. 2016). When NPs are fullerene, it was shown that HSA binds at the interfacial cavity formed by subdomains IIA and IIIA and that this bond is stabilized by van der Waals forces (Li et al. 2015). When investigating other blood proteins like fibrinogen, it was reported that on metal oxide NPs (TiO_2_, CeO_2_, Al_2_O_3_ and ZnO), fibrinogen interacted with TiO_2_ and CeO_2_ NPs with high affinity, while with Al_2_O_3_, the affinity was low and in an 1:1 ratio type. In contrast, HSA interacted with TiO_2_, CeO_2_ and Al_2_O_3_ NPs with low affinity according to a conformational change model. Moreover, fibrinogen associated faster with NPs, while HSA exhibited a lower association pattern (Canoa et al. 2015).

Recent reports that used dynamic light scattering and nanoliquid chromatography tandem mass spectrometry for characterizing the bio-corona formed on lipid and silica NPs have shown that in contact with human plasma, the majority of adsorbed molecules are immunoglobulins, complement factors and coagulation proteins. All these bio-molecules have specific receptors on immune cells which, upon their binding, results in cell activation (Caracciolo et al. 2015). On the other hand, other proteins like albumin and apolipoproteins can inhibit NPs uptake (Cedervall et al. 2007a). Indeed, upon artificial coating of NPs, it was demonstrated that the uptake by macrophages is hindered (Caracciolo et al. 2015).

Higher exchange of IgG in serum proteins adsorbed to the nanostructured surfaces increases structural stability of IgG molecules adsorbed to nanostructured surfaces, while the sterical restrictions imposed by the nanostructure on the binding of IgG affect the interaction with complement proteins (Hulander et al. 2011). The immune complement system is part of the innate immune system, and one of its main functions is to act as a first line of defense against foreign objects; thus, when biomaterials come in contact with blood and other body fluids, complement incompatibility reactions may occur (Nilsson et al. 2007; Remes and Williams 1992). The immune complement is comprised of more than 20 different proteins including activation and inhibition factors. Biomaterials are known agonists of complement and leukocyte activation (Gorbet and Sefton 2004). Complement activation can occur though three pathways upon recognition of a target: classical, alternative and the lectin pathway. Complement activation by any of these pathways results in turnover of the complement protein C3, the production of inflammatory peptides C3a, C4a and C5a, as well as formation of C5b-9 complex or membrane attack complex (Rybak-Smith and Sim 2011; Carroll and Sim 2011). The particle size or concentration at a specific conformational state of grafted polymer does not affect the complement activation on the NP surface. Upon defining the constituents of the protein corona on various NPs, it was shown that the elevated levels of two pro-complement proteins, factors B and C3, are present on the NP surface grafted with glycopolymer chains that are responsible for complement activity (Yu et al. 2014). The classical pathway is activated when the complement protein C1q binds to sufficient number of IgG molecules adsorbed on a surface (Hwang et al. 2008). Complement activation starts concomitantly with the adsorption of the protein film which is triggered by the self-limiting classical pathway activation. After adsorption of the protein film, alternative pathway activation provides the bulk of the C3b deposition that adds 25 % more mass to the surface (Andersson et al. 2005). Although IgG fragments are found in the corona on all particles regardless of size or surface modification, the complement protein C1q is not found on the smaller than 50-nm hydrophilic particles. However, on the unmodified (hydrophobic) particles, where C1q was found on all particles, this phenomenon does not apply (Lundqvist et al. 2008). The C1q–IgG interaction activates the classical complement pathway leading to the generation of terminal membrane attack complex (MAC) (Arlaud et al. 2002). The structure of the IgG binding domain of C1q consists of six globular heads that are equally distributed in a circle of approximately 20 nm in diameter (Kilchherr et al. 1985). Thus, in order to activate C1q, the required number of IgG molecules must be present within this diameter (Hulander et al. 2011; Gaboriaud et al. 2003). As the innate immune system plays a critical role in the protection against NPs, complement activation by nanotubes is consistent with adjuvant effects and might also promote damaging effects of excessive complement activation. C1q binds directly to carbon nanotubes. On the other hand, the protein binding to carbon nanotubes is highly selective. Thus, fibrinogen and apolipoproteins bound to carbon nanotubes in greatest quantity (Salvador-Morales et al. 2006). The adhesion of functionalized carbon nanotubes to phagocytic cells and red blood cells may be altered by the interaction with complement system proteins. However, excessive activation of complement can also cause harmful effects on adjacent human tissues (Rybak-Smith and Sim 2011). “*Playing with the structure”* in context of complement activation was accomplished by studying the roles of polystyrene NPs types in the initiation of the complement cascade. Interestingly, alteration of copolymer architecture on nanospheres from “mushroom” to “brush” configuration not only switched complement activation from the C1q-dependent classical pathway to the so-called lectin pathway, but also reduced the level of generated complement activation products (Hamad et al. 2010).

### Bio-corona inducing acquired immunity

Actually, innate and adaptive immune responses work in harmony to provide efficient protection against invasion by foreign elements, not only by pathogens, but also by nanomaterials (Dumortier 2013). Innate immunity will shape the long-lived adaptive immune response mediated by T and B lymphocytes. PRR from the Toll-like receptor family is one of the main innate immune molecules influencing adaptive immunity. This is orchestrated by DCs that effectively present antigen to naïve T cells (Liu et al. 2013).

Subsequent to entrance into the body, nanomaterials encounter the immune system and may induce desirable or undesirable immunological effects (Dumortier 2013). In this respect, innate immune responses facilitate the participation of adaptive immune responses allowing the body to readily recognize damaged self-macromolecules, thereby increasing the efficiency of elimination. On the other hand, the adaptive immune system can produce a variety of signaling mediators which can stimulate and increase the effectiveness of the innate immune response (Wang et al. 2013). Macrophages express opsonic and a range of non-opsonic receptors, Toll-like receptors (TLRs), retinoic acid-inducible gene I-like (RIG-I-like) and nucleotide-binding oligomerization domain-like (Nod-like) sensing receptors, on their surface as well as in vacuolar and cytosolic compartments. In macrophage activation: the first stage, differentiation, depends on growth factors; the second, priming by interferon-gamma (IFN-γ) during the classical activation of macrophages or IL-4 and IL-13 during the alternative activation of macrophages; and the third stage, a localizing stimulus delivered by a TLR or analogous receptor. Exposure to metal oxide NPs or carbon nanotubes result in a Th1 immune cell microenvironment that promotes the polarization of classically activated macrophages (Gordon and Martinez 2010). Eventually, NPs could modify macrophage phenotype (Ma et al. 2011). Thus, different metallic NPs may regulate innate and adaptive immunity in different directions by modulating dendritic cell functions. While some NPs potentiate human dendritic cells maturation which favors Th-1 responses, the others may promote the secretion of anti-inflammatory cytokines by inducing antigen-presenting cells, leading to Th-2-dominated T cell profile (Schanen et al. 2013). This pronounced T helper response polarization resulting from metal oxide NPs could possibly be due to differences in the capacities of the NP species to regulate reactive oxygen species (ROS) production. ROS are involved as the central second messengers, acting at multiple levels in a multitude of the signaling pathways that regulate the pro-inflammatory genes (Øvrevik et al. 2015). An increase in ROS production by NPs is an initiating step which has the capability to trigger an innate immune response through the activation of the inflammasome (Wang et al. 2013). On the other hand, the key immunological responses related to multi-walled carbon nanotube exposure is NF-kB activation, leading to induction of ROS, thus resulting in the synergistic effect of systemic and local inflammation (Vitkina et al. 2016). Furthermore, metallo-fullerenol NPs can inhibit the growth of tumors by polarizing the cytokine balance toward Th-1 type. Eventually, these particles trigger the host immune system by decreasing the production of Th-2 cytokines and increasing the production of Th1 type cytokines (Liu et al. 2009).

Nanoparticles can definitely influence adaptive and innate immunity through their dynamic bio-corona, but there are still issues that need further clarifications, one of them being the pattern that guides adaptive immunity to trigger immunological memory.

## Nanomedicine applications based on manipulating bio-corona

Nanomedicine applications together with nanopharmaceutics benefit from the rapidly advancing nanotechnology. Nanomedicine research and state-of-the-art advances in nanobiotechnology are closely linked. Research advances have brought high-tech nanoscale measurement that has facilitated the investigation of cellular biological processes; these investigations open the door for nanomedicine (Heico et al. 2012, *NanoImpactNet*). Current research in the field of nanomedicine is focused on how modulating the formation of bio-corona in complex biological environments can result in obtaining more targeted nanodrugs and in decreasing rapid recognition by mononuclear phagocyte system (MPS) and the elimination of the NPs from the body.

Engineered NPs paired with a bio-corona have already been utilized both in in vitro and in vivo rodent models for pharmacokinetic purposes. However, in order to extrapolate these findings to human clinical trials, many physiological factors must be seriously considered, such as the protein nature of the corona, the species used for in vivo evaluation, their basal metabolic rate, circulation blood levels and their size. Furthermore, the importance of the surface area of the body has already been demonstrated, showing the allometric relation of the conjugation and the cellular uptake (Eliasof et al. 2013). Based on this theory, several issues are raised regarding the distribution and the evolution of NPs–bio-corona complex inside the human body. In comparison with the animal models, the human’s body size and the circulation time needed are different; hence, extrapolating data from animal models to humans needs a cautious approach (Sahneh et al. 2015).

Nanomedicines are designed to interact with biological systems at the nanoscale. Thus, the physicochemical properties of nanomaterials play important role as scaffolds for the bio-nanointerface. Bio-corona cannot be ignored in the design of nanomedicines, especially when they are composed of targeting ligands. Moreover, bio-coronas can enhance the effectiveness of NPs used as drug delivery vehicles by increasing their payload capacity (Hamad-Schifferli 2015).

Sanchez-Moreno et al. (2015) studied in detail how the NP uptake and the therapeutic efficacy of the NPs are affected by adding a poloxamer in order to control corona formation. Four-lipid core–shell nanosystem with a hydrophobic core constituted by olive oil and hydrophilic shell by different composition (Pluronic F127 and Epikuron 145 V) was reported. The nature and the concentration of the surfactant can control the interactions between lipid NPs and the biological environment. The blood circulation time of the NPs could be increased by adding a coating, like poloxomer, which leads to a lower uptake by macrophage through reducing unspecific binding, but this likewise attenuated the uptake into the target cells and decreased therapeutic efficacy (Sanchez-Moreno et al. 2015). In order to increase the recognition and uptake at the target sites, targeting moieties could be conjugated on these NPs (Rata-Aguilar et al. 2012; Sanchez-Moreno et al. 2013).

Interactions with serum proteins endow NPs with the best bio-corona for nanomedicine applications (Lynch and Dawson 2008; Mahmoudi et al. 2011a, b). Yallapu et al. (2015) showed that different adsorption patterns dictate the intended target. Apolipoprotein E (apoE) binding to magnetic nanoparticles (MNPs) led to enhanced transport across the blood–brain barrier. Protein binding to a lesser extent resulted in NPs targeting blood circulation, while an increased binding guided NPs toward liver and kidney. The same authors demonstrated that after the protein corona formation, MNPs undergo an uptake process that has an increased dynamics or an increased interaction with the membrane structures, enhancing the overall internalization process in cancer cell lines (Yallapu et al. 2015).

Human serum albumin and apoE are used to steadily form conjugates with polyelectrolyte multilayer-coated gold NP (AuNP) prior to intravenous injection. This coating influences the bio-corona formation and the NP bio-kinetics. Albumin NPs exhibited increased accumulation in lungs, spleen and brain in comparison with the control cit-AuNP. Protein conjugation reduces also liver retention (Schäffler et al. 2014). The application of these findings may overcome some obstacles related to lung-directed therapies and may present a starting point for the design of nanodrugs targeting tuberculosis and other lung diseases. They may also prove useful in designing nanoparticulate drugs intended to cross blood–brain barrier by manipulating the formation of bio-corona in vivo. Indeed, apolipoproteins conjugated with NPs promote the interaction between the NP with low-density lipoprotein receptors leading to an enhanced transport across the blood–brain barrier (Monopoli et al. 2011).

It is well established that the protein corona is affected by several factors including physiochemical properties of the NPs, temperature (Mahmoudi et al. 2013), protein concentration (Caracciolo et al. 2011; Monopoli et al. 2011), incubation time (Tenzer et al. 2013; Lundqvist 2013; Barran Berdon et al. 2013), as well as the protein source (serum vs plasma) or by animal source [mouse plasma (MP) vs human plasma (HP), etc.]. Thus, Caracciolo et al. (2014a, b) showed that MP liposomes are more enriched in apolipoproteins, less enriched in opsonins and more negatively charged than HP liposomes. These findings suggest a different pharmacokinetic profile of liposomes in the bloodstream of humans compared to mice. Hajipour et al. (2014) have recently demonstrated that the pathological changes in different diseases can cause the formation of different protein corona. Thus, the concept of a “personalized protein corona” is a determinant factor for biological and clinical applications. In another study, it has been shown that the cationic liposome–protein corona presented differences in zeta potential between healthy volunteers and histologically proven pancreatic cancer patients, with statistically significant reduction in the level of clinically relevant proteins (Caracciolo et al. 2014b). All these findings led to the concept of manipulating the formation of protein corona to get targeted drugs and gene delivery using liposomes. The design of liposomes can provide the uptake of proteins in bio-corona that are specifically recognized by receptors expressed on target cells. Yokoe et al. (2008) showed that when albumin is conjugated to drug-loaded PEGylated liposomes, their blood circulation is enhanced along with their superior therapeutical action. These authors thus demonstrated that albumin decreased opsonin binding to PEGylated liposomes and that the therapeutic activity against sarcoma of doxorubicin-loaded liposomes is improved (Yokoe et al. 2008). A separate study showed that the serum proteins enhanced the binding of cationic NPs to the cells and inhibited the binding of anionic NP s. This behavior depends on cellular receptors targeted by these complexes between serum protein and NPs (Fleischer and Payne 2012).

Moreover, in regard to further biomedical approaches, the bio-nanocorona complex could open new horizons for research on the bio-nanointeractions, such as the interactions with the cells of the innate and the adaptive immune system. As recently published, the engineered bio-corona NPs could prove to be very helpful tool for immunomodulation, supporting fertile ground for better engineering and advanced design of immunologically safer target nanosystems. Specifically, Fang et al. (2014) revealed a whole new delivery *scenario*, by designing a bio-complex of NPs to mimic red blood cells and thus to escape from the immune system. They combined biodegradable polymeric NPs with membrane lipids and membranes derived from blood cells and transformed them into efficient bio-carriers. Results indicated that even the delivery and the release of slowly biodegradable drugs had a better uptake with the use of bi-mimetic carriers, as compared to the standard PEG “pathway” (Fadeel et al. 2010; Nystrom and Fadeel 2012).

Another application with good future perspectives is vaccination. We have discussed in the previous sections that nanomaterials interact with innate immune cells through TLRs but as well as with other immune molecular systems such as the complement system. Nanomaterials that carry the complex bio-corona can activate the inflammasome in macrophages leading to pro-inflammatory cytokines cascade. Importantly, these findings can be manipulated in developing novel classes of immunostimulatory agents (Hubbell et al. 2009). Cells of the innate immune arm can enzymatically digest nanomaterials based on carbon materials, resulting thus in materials that are cleared off from the organism and that are not persistent as asbestos fibers. Therefore, one can imagine that carbon nanotubes can be good carriers for therapeutic agent’s delivery (Fadeel 2012). The bio-corona on NP’s surfaces critically discriminates the biological responses and hence the involvement in any therapeutical approach. Danger signals described above and recently reviewed in bio-corona context (Farrera and Fadeel 2015) can have therapeutically positive effects. Knowing that vaccine adjuvants (alum) used since the 1920s (Mbow et al. 2011) enhance the immunogenicity of a co-administered antigen (De Gregorio et al. 2008) can shed new light on nanomaterials applications that could be extended as adjuvant carriers and/or deliver *per se* other adjuvants (Hubbell et al. 2009). Using pluronic-stabilized polypropylene sulfide (PPS) NPs conjugated to the model antigen OVA, the generation of humoral and cellular immunity (Reddy et al. 2007) was demonstrated. In this model, the complement cascade was generated, engendering a “danger” signal that activated DCs. A typical vaccination reaction was obtained hence. Mice immunization with synthetic NP, poly(d, l-lactic-co-glycolic acid) (PLGA) containing antigen, plus ligands that activate TLR4 and TLR7 can induce higher titers of antigen-specific, neutralizing antibodies compared to immunization with NPs specific for just one TLR. This model proved its usefulness in protecting against lethal avian and swine influenza virus strains. This vaccination type was also tested in non-human primates that show a different TLR7 expression on DCs as we compared to murine strains (Kasturi et al. 2011). This vaccine resembling a virus both in size and in composition recapitulated the immunogenicity of live viral vaccines. Later on, a new NP-based toxin detainment was used for delivering pore-forming toxins. Mice vaccinated with the NP-detained toxin displayed superior protective immunity (Hu et al. 2013). Engineered NPs can go as far as artificial antigen-presenting cells, delivering antigen plus immunostimulatory molecules for vaccination. NPs can mimic microorganisms like viruses, surpassing all the complications of recombinant viral vaccines (Moon et al. 2011, 2012). Delivering antigens to antigen-presenting cells was reported several years ago (Dumortier 2013; Villa et al. 2011). Single-walled carbon nanotubes were used as carriers for delivery of peptides into antigen-presenting cells and were able to induce IgGs against weak tumor-associated antigens. More recently, the utilization of PEG-modified single-walled carbon nanotubes armed with the glucocorticoid-induced TNFR-related receptor (GITR) demonstrated in vivo targeting of regulatory T cells residing in a B16 melanoma in mice (Sacchetti et al. 2013).

It is well established that certain small molecules, non-immunogenic haptens, can elicit an immune response when coupled to a protein carrier (Boraschi et al. 2012). Thus, immunization of mice with a C60 fullerene derivative conjugated to bovine thyroglobulin yielded IgG antibodies specific to fullerenes (Chen et al. 1998). Different classes of NPs are promising tools for immunomodulation, but their long-term safety needs to be examined in the course to clinical translation. Nevertheless, NPs’ bio-corona can induce specific immune responses, which presents further biological ground for future nanomedicine applications.

## Toxicological particularities of NPs’ protein bio-corona

Several aspects within this section were touched upon in the previous sections, but some final highlights of the toxicological aspects that are raised by the protein bio-corona need to be resolved. Due to NPs’ physical characteristics and high surface area–volume ratio, there remain unknown toxicity mechanisms within the biological systems that remain to be unraveled (Oberdorster 2010). Several recent studies have shown that bio-corona clearly induces cellular responses. Thus, p38 MAPKs which are stress-activated kinases are recently shown to be induced by positively charged mesoporous silica NPs, whereas negatively charged NPs induce ROS. However, when NPs were coated with proteins, their cellular deleterious effect was significantly reduced (Liu et al. 2015). Inflammatory responses, portrayed by the activation of IL-6, can be induced by AgNPs with or without a protein corona (Shannahan et al. 2015). As discussed in the previous sections, single-walled carbon nanotubes bind different blood proteins and were shown to induce different cytotoxicity issues (Ge et al. 2011), while graphene oxide covered with a protein corona was less cytotoxic compared to naked ones (Hu et al. 2011). There is a turn in the story when we analyze the immune response developed by bio-corona covered NPs. Proteins that comprise the bio-corona, IgG, complement, albumins, lipoproteins, and so on, can generate cellular immune response leading up to inflammation (Sim and Wallis 2011). Overcoming this impediment relies on a controlled NP’s surface composition and hence controlled biomolecules absorption (Hulander et al. 2011; Bertholon et al. 2006).

As indicated above, the bio-corona is a dynamic entity and the toxicological impact of it needs to be determined by unveiling toxicologically relevant phenomena at the nano-bio-interface (Westmeier et al. 2015). Concentrating on the future of nanotoxicology in this framework, Westmeier et al. (2015) have outlined some important directions to follow. Thus, highly advanced in vitro cellular models should reproduce the major entry routes of NPs to biological systems, in order to sustain high-throughput analysis and standardization. Another modality important in resolving the toxicological issues for inter-cellular communication, intra-/inter-cellular signaling pathways and complex responses to NPs is to go up to organ-on-a chip (Lancaster et al. 2013; Nguyen et al. 2013; Westmeier et al. 2015). In vitro testing should advance to in vivo models where predictability of NPs’ influence on human health needs to be examined (Nel et al. 2013). Although there are various NPs toxicology animal models, a highly specific animal model for bio-corona research is still to be developed (Westmeier et al. 2015). Moreover, it must be stressed that in nanotoxicology issues, the mere evaluation of cell’s viability is not sensitive enough to unveil the complex processes of bio-corona formation in vivo and hence to predict NPs fate in a complex biological system.

## Conclusion

Understanding the complex interaction of NPs with the biological microenvironments of the host organism and the resulting biological actions is one of the goals of nanotoxicology and the nanomedicine field. All the physicochemical properties that characterize NPs alter the immune interactions. Specific adsorption of biomolecules bestows onto the NP a new immunological identity (Fadeel 2012). Understanding the subtle kinetics of the bio-corona formation process can be seminal in predicting NP behavior in biological systems, including applications of nanotoxicology and development of nanodrug delivery platforms. As NPs are in the size range of biological aggressors, interactions with the immune system are more likely to occur (Dobrovolskaia and McNeil 2007; Andón and Fadeel 2013; Shvedova et al. 2010). Indeed, immune recognition of microbial molecules (Kono and Rock 2008) can be activated in NPs immune recognition. In this light, nanomaterials able to interact with the immune cells are of utmost importance in terms of biological activity (Shvedova et al. 2010). Noteworthily, NPs entering the organism develop a specific bio-corona comprising complex and dynamic layers of biomolecules that endow NPs with a new immunological identity. Understanding how the nanomaterial interacts with its biological surroundings is of utmost importance, and its application can specifically target novel nanodrugs not only at the systemic scale, but as well as to the level of intra-cellular compartments (Constantin et al. 2010; Socoteanu et al. 2015). In this light, engineered nanomaterials can be tailored for “smart” drug delivery with applications that start from in vivo imaging and go to regenerative medicine.

Pinpointing individual nanomaterial characteristics (Fadeel et al. 2013) can foresee the biological and medical fate of the nanodrug (Fadeel et al. 2015). NPs can cross biological barriers, thus making them perfect drug delivery systems, but the further fate of NPs, e.g., excretion, biodegradation, accumulation or any other long-term non-immune/immune processes, should be clearly understood while developing safer nanomedicines (Nystrom and Fadeel 2012).
